# Dispersion of Few-Layer Black Phosphorus in Binary Polymer Blend and Block Copolymer Matrices

**DOI:** 10.3390/nano11081996

**Published:** 2021-08-03

**Authors:** Serena Coiai, Elisa Passaglia, Simone Pinna, Stefano Legnaioli, Silvia Borsacchi, Franco Dinelli, Anna Maria Ferretti, Maria Caporali, Manuel Serrano-Ruiz, Maurizio Peruzzini, Francesca Cicogna

**Affiliations:** 1 CNR-ICCOM, Consiglio Nazionale delle Ricerche, Istituto di Chimica dei Composti OrganoMetallici, SS Pisa, Via Moruzzi 1, 56124 Pisa, Italy; elisa.passaglia@pi.iccom.cnr.it (E.P.); simop93@hotmail.it (S.P.); stefano.legnaioli@pi.iccom.cnr.it (S.L.); silvia.borsacchi@pi.iccom.cnr.it (S.B.); francesca.cicogna@pi.iccom.cnr.it (F.C.); 2 CISUP, Centro per l’Integrazione della Strumentazione Scientifica dell’Università di Pisa, 56126 Pisa, Italy; 3 CNR-INO, Consiglio Nazionale delle Ricerche, Istituto Nazionale di Ottica, Via Moruzzi 1, 56124 Pisa, Italy; franco.dinelli@ino.cnr.it; 4 CNR-SCITEC, Consiglio Nazionale delle Ricerche, Istituto di Scienze e Tecnologie Chimiche, Via G. Fantoli 16/15, 20138 Milano, Italy; anna.ferretti@scitec.cnr.it; 5 CNR-ICCOM, Consiglio Nazionale delle Ricerche, Istituto di Chimica dei Composti OrganoMetallici, Via Madonna del Piano 10, 50019 Sesto Fiorentino (FI), Italy; maria.caporali@iccom.cnr.it (M.C.); manuel.serrano@iccom.cnr.it (M.S.-R.); maurizio.peruzzini@iccom.cnr.it (M.P.)

**Keywords:** few-layer black phosphorus, polymer blend composites, in situ RAFT polymerization, morphology

## Abstract

Exfoliated black phosphorus (bP) embedded into a polymer is preserved from oxidation, is stable to air, light, and humidity, and can be further processed into devices without degrading its properties. Most of the examples of exfoliated bP/polymer composites involve a single polymer matrix. Herein, we report the preparation of biphasic polystyrene/poly(methyl methacrylate) (50/50 wt.%) composites containing few-layer black phosphorus (fl-bP) (0.6–1 wt.%) produced by sonicated-assisted liquid-phase exfoliation. Micro-Raman spectroscopy confirmed the integrity of fl-bP, while scanning electron microscopy evidenced the influence of fl-bP into the coalescence of polymeric phases. Furthermore, the topography of thin films analyzed by atomic force microscopy confirmed the effect of fl-bP into the PS dewetting, and the selective PS etching of thin films revealed the presence of fl-bP flakes. Finally, a block copolymer/fl-bP composite (1.2 wt.%) was prepared via in situ reversible addition–fragmentation chain transfer (RAFT) polymerization by sonication-assisted exfoliation of bP into styrene. For this sample, ^31^P solid-state NMR and Raman spectroscopy confirmed an excellent preservation of bP structure.

## 1. Introduction

The recent attention given to black phosphorus (bP), an anisotropic layered p-type semiconductor material, is essentially due to the outstanding electronic properties of its two-dimensional (2D) form known as phosphorene or 2D-phosphane [1,2,3,4,5,6]. Exfoliated bP has a thickness-dependent tunable direct bandgap, which varies from 0.3 eV (bulk) to 2 eV (monolayer), anisotropic transport properties, high charge carrier mobility, high conductivity, and good current saturation in field-effect devices [7,8]. Owing to these fascinating properties, both monolayer and few-layer forms of bP are attracting a continuously growing interest for applications in optoelectronics and electronics, photocatalysis, photonic, medical, and thermoelectric devices [9,10,11,12,13,14,15,16]. On the other hand, a few factors still limit the full exploitation of the technological potential of phosphorene. In particular, bP is much harder to exfoliate as compared to other precursors of 2D materials, and it is quite difficult to obtain large-area sheets with a controllable thickness [17,18]. bP can be mechanically exfoliated into ultrathin films by repetitive cleaving of a bulk crystal using an adhesive tape [18] even if the method is not scalable and is generally used only for research purposes. Liquid phase exfoliation (LPE) of bulk bP is an alternative method for the large-scale preparation of bP nanosheets. Different solvents have been used to exfoliate the bP, such as *N*-methyl-2-pyrrolidone (NMP), *N*-cyclohexyl-2-pyrrolidone (CHP), and dimethyl sulfoxide (DMSO) [19,20,21]. Monolayer and few-layer bP (fl-bP) are environmentally unstable because of their high reactivity to oxygen, water, and light, which cause undesired reactions degrading the material and altering its chemical–physical properties [22]. Interesting advances about fl-bP stability have been achieved by encapsulation into a polymer matrix. This method represents an effective way to prevent fl-bP degradation by oxidation [23] and improve the dispersion, as already proved for other 2D-precursor materials, such as layered clays [24]. Moreover, polymers, in particular, thermoplastic polymers, are attractive supports, because they can be easily processed and fabricated into solid-state forms such as thin films, as often required in most sensor applications.

To date, only a few articles describe the preparation of polymer/fl-bP composites due to the instability of fl-bP and demanding procedures required to exfoliate bP in scalable amounts. Del Rio Castillo et al. [25] reported the dispersion of exfoliated and functionalized flakes of bP into poly(methyl methacrylate) (PMMA), showing an increase in Young’s modulus. Ni et al. [26] described the encapsulation of bP nanosheets into poly(vinyl alcohol) (PVA) obtaining reinforced and air-stable PVA nanocomposites. Tiouitchi et al. [27] dispersed exfoliated bP into poly(vinylidene fluoride), evidencing an improvement in rigidity and thermostability. Qiu et al. [28] showed that the inclusion of bP flakes into UV-curable polyurethane acrylate (PUA) improves the flame resistance and mechanical strength of PUA coatings, and, similarly, Ren et al. [29] demonstrated the flame resistance of phosphorene-waterborne polyurethane composites. Furthermore, He et al. [30] described the exfoliation of bP by electrochemical cathodic method with simultaneous modification of flake surface. This method improves the compatibility between bP and waterborne polyurethane matrix by improving the mechanical properties and flame retardancy. Furthermore, a very efficient flame retardant for this type of composite was obtained by combining bP and boron nitride [31]. Recently, it has also been shown that bP can improve both the mechanical properties and flame retardancy of epoxy resins [32,33]. Advances have been achieved through functionalization of bP by adsorption of organic molecules [34] or surface coordination of metal ligands [35,36]. For example, bP was coordinated with a ruthenium sulfonate and a lanthanide metal ligand, respectively, achieving excellent stability in ambient conditions and common solvents, and increasing the dispersibility in epoxy resin. The functionalized bP/epoxy resin composites showed a significant improvement in flame retardancy and thermal conductivity. In addition, the synergistic effect of bP and multi-walled carbon nanotubes (MCNTs) further improved the flame retardancy of epoxy resin [37].

We have recently investigated the incorporation of fl-bP flakes in a polymer matrix by following two different approaches: solution blending and in situ polymerization [38,39]. With the first approach, we demonstrated that fl-bP embedded into polystyrene (PS) or PMMA is preserved from oxidation, is stable to air, light, and humidity, and can be processed into devices without degrading its properties. Furthermore, the samples showed improved thermal stability compared to neat polymer and were resistant to photo-degradation. The in situ polymerization was used to promote further exfoliation and dispersion of the stacked phosphorene layers due to the energy developed during polymerization. In addition, this method provides intimate contact between the nanosheets and polymer chains growing in proximity. Again, efficient devices were fabricated [40].

The incorporation of 2D nanoparticles in polymer blends or block copolymers is a strategy adopted to promote the selective distribution of nanoparticles in one of the two phases or at the interfacial region [41,42]. New collective properties can emerge from the confinement of nanoparticles in a narrow region. In addition, in the case of co-continuous morphology of the polymer matrix, nanoparticles can arrange in percolation paths incrementing specific properties such as conductivity and barrier features. In immiscible binary polymer blends, for example, nanoparticles can be selectively distributed in one of the two phases or at the interface depending on their size, shape, and chemical characteristics enabling the preferential interaction with one of the two polymers [41]. Gelfer et al. [43] reported the preferential location of clay layers in the PMMA phase of PS/PMMA (50/50 wt.%) blend due to the match in polarity. This distribution increases the viscosity of the PMMA phase and reduces the PS domain size. Mao et al. [44] investigated the dispersion of graphene nanosheets in PS/PMMA (50/50 wt.%) blend, showing that 0.5 wt.% of functionalized graphene provided the formation of a co-continuous structure of PS and PMMA phases and that graphene nanosheets were selectively located and percolated in the PS phase. In this condition, a conductive composite with a low electrical percolation threshold was obtained. Similarly, Bai et al. [45] demonstrated that reduced graphene oxide could be trapped in the form of multilayers at the interface of co-continuous poly(lactic acid)/PS blends, stabilizing the morphology and increasing the conductivity with a low electrical percolation threshold. 

The dispersion of bP into a block copolymer with a well-ordered microstructure is also an interesting method to obtain new materials with specific functional properties. These materials can find applications in several sectors such as catalysis, sensors, and electronic devices, as demonstrated for other types of nanoparticles [42,46]. The block copolymer can be used as a template to sequester the nanoparticles into a particular region of the matrix, as well as to orient them by the desired application. Most examples refer to 0D metal nanoparticles or quantum dots with length scales smaller than the dimensions of the block copolymer microstructure. However, sheet-like nanoscale fillers have also been added to block copolymers. Exfoliated clay sheets, for example, were embedded into a styrene-ethylene/butylene-styrene triblock copolymer, evidencing a preferential wetting of the sheets by the PS block [47]. Furthermore, it was demonstrated that exfoliated clay sheets influence the orientation of block copolymer lamellae due to their size and shape relative to that of the microdomains. 

**Figure 1:**
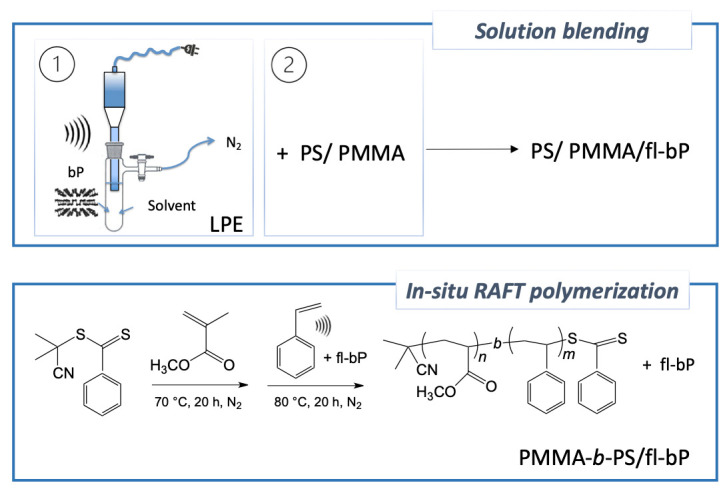
Schematic depiction of proposed methods of preparation of fl-bP polymer composites: solution blending and in situ RAFT polymerization.

To the best of our knowledge, the embedding of fl-bP into immiscible polymer blends or block copolymers has not been investigated yet. Therefore, we report the dispersion of fl-bP into a PS/PMMA blend (50/50 wt.%) to investigate the influence of fl-bP on the morphology of the blend, the possible arrangement of fl-bP at the interphase, or its preferential segregation in one of the two phases. Furthermore, we describe for the first time the preparation of a block copolymer/fl-bP nanocomposite (PMMA-*b*-PS/fl-bP) via in situ reversible addition–fragmentation chain transfer (RAFT) polymerization with direct sonication-assisted exfoliation of bP into styrene (Figure 1). Micro-Raman and ^31^P solid-state NMR (SSNMR) spectroscopies are used to investigate the integrity and the distribution of fl-bP, while the morphology is studied by optical microscopy, scanning electron microscopy (SEM), transmission electron microscopy (TEM), and atomic force microscopy (AFM). Thermal properties are also measured by thermo-gravimetric analysis (TGA) and differential scanning calorimetry (DSC) to provide information about the thermal stability of composites and thermal transitions of polymer phases.

## 2. Materials and Methods

### 2.1. Materials

Styrene (Sty) ≥99% and methyl methacrylate (MMA) 99% (Sigma-Aldrich, Schnelldorf, Germany) were vacuum-distilled and stored under N_2_. The 2,2′-azobis(2-methylpropionitrile) (AIBN) 98% and 2-cyano-2-propyl benzodithioate (CPDB) (Sigma-Aldrich, Schnelldorf, Germany) were used as received. The solvents *N*-cyclohexyl-2-pyrrolidone (CHP) 99%, toluene ≥99.7%, cyclohexane ≥99.5%, chloroform 99.0–99.4%, methanol ≥99.9%, 2-propanol ≥99.5%, ethanol absolute ≥99.8%, acetone ≥99.6%, and glacial acetic acid ACS for analysis (Sigma-Aldrich, Schnelldorf, Germany) were used as received. bP crystals were prepared according to the procedure developed by Köpf et al. as previously described [48]. General-purpose polystyrene (PS), also known as crystal polystyrene with *M_n_* = 90,000 g/mol (Repsol, Madrid, Spain), and poly(methyl methacrylate) (PMMA) with *M_n_* = 120,000 g/mol (Sigma-Aldrich, Schnelldorf, Germany) were used as obtained. Boron-doped silicon wafers with oxide layer of 300 nm, <100> orientation, resistivity 1–5 Ω·cm, single side polished (ssp), squared slides with size 15 × 15 mm^2^, and 675 ± 25 μm thickness (MicroFabSolutions S.r.l., Trento, Italy) were used after ultrasonic cleaning in acetone for 5 min and, then, in acetone and 2-propanol for an additional 5 min each.

### 2.2. Preparation of PS/PMMA/fl-bP Composites by Solution Blending

#### 2.2.1. bP Exfoliation

In a typical procedure, 10 mg of bulk bP were weighed and crushed under a nitrogen atmosphere and, then, put into a test tube containing 10 mL of degassed CHP. The suspension was probe-sonicated under nitrogen flow for 8 h using an Ultrasonic Processor UP200St (200 W, 26 kHz) (Hielscher Ultrasonics GmbH, Teltow, Germany) equipped with a titanium 2 mm sonotrode (S26d2), which was used at 50% of the maximum amplitude at an effective power density of 4 W·cm^−2^. The temperature inside the tube was kept low by using an ice-water bath. After exfoliation, the suspension was used as it is (fl-bP0, 1 mg/mL), or after centrifugation at 3000 and 5000 rpm, respectively, for 20 min, thus separating small flakes in the supernatant from larger ones in the sediment. In both cases, the supernatants, fl-bP3000 and fl-bP5000, were collected and analyzed with dynamic light scattering (DLS). The quantity of material settled on the bottom of the tube after centrifugation at 3000 and 5000 rpm was roughly determined by weighing, and it resulted in about 50% and 70% of the initial quantity of bP. Accordingly, the concentration of flakes in fl-bP3000 was 0.5 mg/mL whereas that of fl-bP5000 was 0.3 mg/mL. The average hydrodynamic diameter of flakes by DLS analysis was 358 ± 56 nm for fl-bP0, 270 ± 26 nm, and 212 ± 24 nm for fl-bP3000 and fl-bP5000, respectively. A summary of fl-bP suspensions is provided in Table S1.

#### 2.2.2. Preparation of PS/PMMA/fl-bP Composites

PS/PMMA ratio of 50/50 wt.% was selected. For comparison purposes, a blank sample of PS/PMMA was prepared by introducing 250 mg of PMMA and 10 mL of CHCl_3_ into a 100 mL two-necked round-bottom flask, equipped with a magnetic stirrer and backfilled three times with nitrogen. The solution was magnetically stirred in a continuous stream of nitrogen until polymers were completely dissolved. Then, under nitrogen flow, 5 mL of CHP were added to the PS/PMMA solution, and the mixture was left stirring for 15 min. Finally, the polymer blend was recovered by precipitation in methanol, and the solid was filtered and dried in a vacuum oven at 45 °C until constant weight. Three PS/PMMA/fl-bP composites (i.e., PS/PMMA/fl-bP0, PS/PMMA/fl-bP3000, and PS/PMMA/fl-bP5000) were prepared by keeping constant the PS/PMMA ratio (50/50 wt.%). The sample PS/PMMA/fl-bP0 was prepared by introducing 250 mg of PS and 250 mg of PMMA and 10 mL of CHCl_3_ into a 100 mL two-necked round-bottom flask, equipped with a magnetic stirrer and backfilled three times with nitrogen. The solution was magnetically stirred in a continuous stream of nitrogen until polymers were completely dissolved. Then, under nitrogen flow, 5 mL of the suspension fl-bP0 (1 mg/mL) in CHP, obtained after sonication, were added dropwise to the PS/PMMA solution and the mixture was left stirring for 15 min. Finally, PS/PMMA/fl-bP0 nominally containing 1 wt.% of fl-bP0 was recovered by precipitation in methanol, and the solid was filtered and dried in a vacuum oven at 45 °C until constant weight. The samples PS/PMMA/fl-bP3000 and PS/PMMA/fl-bP5000 were prepared with the following procedure: into a 50 mL two-necked round-bottom flask, equipped with a magnetic stirrer and backfilled three times with nitrogen, were introduced 125 mg of PS, 125 mg of PMMA, and 5 mL of CHCl_3_. The solution was magnetically stirred in a continuous stream of nitrogen until polymers were completely dissolved. Then, under nitrogen flow, 5 mL of CHP suspension fl-bP3000 (0.5 mg/mL) or 5 mL of CHP suspension bP5000 (0.3 mg/mL) were added dropwise to the PS/PMMA solution and the mixture was left stirring for 15 min. Finally, the composites were recovered by precipitation in methanol, and the solids were filtered and dried in a vacuum oven at 45 °C until constant weight. PS/PMMA/fl-bP3000 and PS/PMMA/fl-bP5000 nominally contain 1 wt.% and 0.6 wt.% of fl-bP3000 and fl-bP5000, respectively. Finally, film samples of 20–30 μm thickness were prepared by compression molding using a Carver press model 4386 (Wabash, IN, USA) preheated at the temperature of 180 °C and used for further analysis. A summary of PS/PMMA/fl-bP composites is provided in Table S1.

### 2.3. Preparation of PMMA-b-PS/fl-bP Composite via In Situ RAFT Polymerization 

The copolymerization was conducted in bulk and in two steps [49]. In the first step, 1.6 mL of MMA (15 mmol), 13 mg of CPDB (0.06 mmol), and 5 mg of AIBN (0.03 mmol) were introduced into a Schlenk tube (10 mL) equipped with a magnetic stirrer and backfilled three times with nitrogen. The stoichiometry of reagents was MMA:CPDB:AIBN = 250:1:0.5. The tube was degassed by three freeze–pump–thaw cycles, left under nitrogen, and then, heated at 70 °C for 20 h. Polymerization was stopped by cooling the content to room temperature and exposing it to air. The PMMA-CPDB (macro-RAFT agent) was purified from the unreacted monomer and polymerization byproducts by dissolving the crude reaction mixture in CHCl_3_ and recovering the polymer by precipitation into a large excess of MeOH. The polymer was filtered, dried in a vacuum oven, and then, analyzed (Table 1). Next, the PMMA-CPDB sample was split in two fractions: one part was used to prepare the block copolymer PMMA-*b*-PS, whereas the second fraction was used to prepare the PMMA-*b*-PS/fl-bP composite (Table 1).

In the case of PMMA-*b*-PS, PMMA-CPDB (500 mg) was dissolved in Sty (1.5 mL, 13.1 mmol) with the addition of AIBN (1.4 mg, 0.008 mmol). The stoichiometry of reagents was Sty:macro-RAFT:AIBN = 780:1:0.5. The theoretical *M_n_* value of the Sty block was 50,000 g/mol. The tube was degassed by three freeze-pump-thaw cycles, left under nitrogen, and then, heated at 80 °C for 20 h. Polymerization was stopped by cooling the content to room temperature and exposing it to air. The block copolymer PMMA-*b*-PS was purified from the unreacted monomer and polymerization byproducts by dissolving the crude reaction mixture in CHCl_3_ and recovering the polymer by precipitation into a large excess of MeOH. The polymer was filtered, dried in a vacuum oven, and then, analyzed (Table 1).

The PMMA-*b*-PS/fl-bP sample was prepared as follows: 10 mg of bP were weighed and crushed under nitrogen atmosphere and, then, put into a test tube containing 1.5 mL of degassed Sty (13.1 mmol). The suspension was probe-sonicated under nitrogen flow for 90 min using a Hielscher Ultrasonic Processor UP200St (200 W, 26 kHz) equipped with a titanium 2 mm sonotrode (S26d2), which was used at 50% of the maximum amplitude at an effective power density of 4 W·cm^−2^. The temperature inside the tube was kept low by using an ice-water bath. The average hydrodynamic diameter of flakes by DLS analysis was 550 ± 80 nm. Then, 500 mg of PMMA-CPDB and 1.4 mg of AIBN (0.008 mmol) were introduced into the tube. The tube was degassed by three freeze–pump–thaw cycles, left under nitrogen, and then, heated at 80 °C for 20 h. The product PMMA-*b*-PS/fl-bP was purified from the unreacted monomer and polymerization byproducts by dissolving the crude reaction mixture in CHCl_3_ and recovering the polymer composite by precipitation into a large excess of MeOH. The composite was filtered, dried in a vacuum oven, and then, analyzed (Table 1). Considering the polymerization yield shown in Table 1 and assuming that all of the fed bP remained in the composite during the preparation steps, PMMA-*b*-PS/fl-bP contains approximately 1.2% fl-bP by weight. A summary of samples prepared by RAFT polymerization is provided in Table S1.

### 2.4. Characterization 

Dynamic light scattering (DLS) measurements were performed with a NanoZetaSizer apparatus model: ZEN1600 (Malvern, Worcestershire, UK) equipped with a HeNe laser (633 nm, 4 mW) and an avalanche photodiode detector with an angle of 173°. The hydrodynamic diameter values and polydispersity index (PDI) were extracted from cumulant analysis and multimodal size distribution algorithm non-negative least square (NNLS). For each measurement, autocorrelation functions were averaged and evaluated by the Dispersion Technology Software (DTS) (Malvern Instruments Ltd., Malvern, UK). 

Raman spectroscopy was performed using an inVia instrument (Renishaw, Wotton-Under-Edge, UK) coupled with an optical Leica DLML microscope (Leica Microsystems, Wetzlar, Germany), equipped with an NPLAN objective 50× (spot size of about 5 μm in diameter). The instrument has an Nd: YAG laser source at *λ* = 532 nm wavelength. The spectrometer consists of a single grating monochromator (1800 lines/mm), coupled with a CCD detector, a RenCam 578 × 400 pixels (22 μm × 22 μm) cooled by a Peltier element. The spectral calibration of the instrument was performed on the 520.5 cm^−1^ band of a silicon wafer. The spectral resolution was 0.5 cm^−1^, and the spectral range was between 100 and 3200 cm^−1^. Polymer samples were analyzed as films obtained by compression molding (as described above) or by spin-coating on a silicon wafer (as described below). To improve the signal-to-noise ratio, the data were accumulated over 3 or more acquisitions with an exposure time of 10 s per acquisition. 

Scanning electron microscopy (SEM) of blends and composites was carried out by using a dual-beam instrument Gaia 3 (TESCAN, Brno, Czech Republic). The elemental analysis on the surface of the samples was carried out by an energy-dispersive X-ray (EDAX) system interfaced with SEM (AMETEK, Mahwah, NJ, USA, software TEAM EDS Basic Suite). The micrographs were acquired on liquid nitrogen fractured films, which were prepared by compression molding, as described above. Both secondary electrons (SE) and backscattered electrons (BSE) detectors were used. Films of samples were also characterized after etching of the PS phase with cyclohexane. For performing this experiment, the fractured surface of the films was immersed in cyclohexane at 60 °C for 7 h. At the end, the film was washed with fresh cyclohexane and, then, dried under vacuum at 50 °C before being analyzed by SEM. 

Atomic force microscopy (AFM) was carried out by using a hybrid system made of a commercial SMENA head (NT-MDT, Moscow, Russia) with electronics and control software developed in-house, and of a digital HF2LI lock-in amplifier (Zurich Instruments, Zurich, Switzerland) to collect the data. AFM has been operated in the so-called tapping mode at ambient temperature and employing the cantilever HQ:NSC35 (MikroMasch, Wetzlar, Germany) with a nominal spring constant of around 5 N·m^−1^ and 150 kHz. The color code of the images is the following: a brighter color corresponds to a higher region. Films of both polymer blends and composites were prepared by spin-casting 60 mL aliquots of a toluene solution (5% wt./vol or 1% wt./vol) under ambient conditions onto a polished silicon wafer, which was rotated at 500 rpm for 30 s and 3000 rpm for 60 s. A WS-400-6NPP-LITE spin processor (Laurel Technologies Corporation, North Wales, PA, USA) was used. The specimens were subsequently annealed at 150 °C in a vacuum oven for 24 h and characterized before and after etching of PS phase with cyclohexane and etching of PMMA phase with acetic acid. The RMS roughness (the root mean square average of height deviations from the mean data plane) and *h*_max_ (the maximum height of the bumps) were measured.

Transmission electron microscopy (TEM) was performed by HR-TEM 200 kV ZEISS LIBRA 200 FE (Carl Zeiss AG, Jena, Germany) equipped with a second-generation in column Ω filter, HAADF detector. The energy dispersive X-ray analysis (EDX) was performed by EDX, OXFORD X-Stream 2, and INCA software (Oxford Instruments Analytical, High Wycombe, Buckinghamshire, UK). The samples were prepared by dropping a toluene solution of PS/PMMA/bP5000 (2 mg/mL) on a lacey carbon copper grid and letting it dry overnight.

Thermogravimetric analysis (TGA) was performed using a SII ETG/DTA 7200 EXSTAR (Seiko instrument, Chiba, Japan). Polymer blends and composites (5–10 mg) were placed in alumina sample pans (70 μL), and runs were carried out at the standard rate of 10 °C·min^−1^ from 30 to 700 °C under nitrogen (200 mL·min^−1^). The onset temperature (*T*_onset_) of the TG curve, calculated as the temperature of intercept of tangents before and after the degradation step, and the temperature of the maximum degradation rate (*T*_mdr_), calculated as the maximum of the peak in the DTG curve, were determined.

Differential scanning calorimetry (DSC) measurements were performed on 5–10 mg samples under nitrogen atmosphere (nitrogen flow was 50 mL·min^−1^ for all the experiments) by using a DSC-4000 differential scanning calorimeter thermal analyzer (Perkin-Elmer, Waltham, MA, USA) equipped with a 3-stage cooler able to reach −130 °C. Previously, the instrument was calibrated by using indium (m.p. 156.6 °C, ΔH = 28.5 J/g) and zinc (m.p. 419.5 °C). Polymer blends and polymer composites were heated from 30 to 180 °C at 10 °C/min (1st heating), cooled to 30 °C at the same scan rate (1st cooling), then, heated again to 180 °C at 10 °C/min (2nd heating). Glass transition temperature (*T*_g_) of both polymer blends and polymer composites was measured from the inflection point in the 2nd heating thermogram.

Size exclusion chromatography (SEC) analysis was carried out by the Agilent Technologies 1260 Series instrument equipped (Agilent Technologies, Santa Clara, CA, USA) with an Agilent degasser, an isocratic high-performance liquid chromatography (HPLC) pump, an Agilent refractive index (RI) detector, one pre-column PLgel 5 μm guard, and two PLgel MiniMIX-D 5 μm columns conditioned at 35 °C. CHCl_3_ was used as the mobile phase at a flow rate of 0.3 mL/min. The system was calibrated with PS standards in a range from 500 to 3 × 10^5^ g/mol. Samples (i.e., PMMA-CPDB, PMMA-*b*-PS, PMMA-*b*-PS/fl-bP) were dissolved in CHCl_3_ (2–4 mg/mL) and filtered through a 0.20 μm syringe filter before analysis. This latter step involves a possible fractionation of the polymer with retention on the filter of recombination chains, as well as partially cross-linked, branched, or entangled polymer chains. However, this step is necessary in the case of composite, because it allows removing from the solution any microaggregates due to the presence of bP-flakes. *M_n_* and *M_w_* were determined using Agilent ChemStation software.

^31^P solid-state NMR (SSNMR) experiments were carried out with an Infinity Plus 400 spectrometer (Varian, Palo Alto, CA, USA) operating at Larmor frequencies of 400.34 and 162.07 MHz for ^1^H and ^31^P nuclei, respectively. Spectra were acquired using a 3.2 mm probe head, exploiting the direct excitation (DE) pulse sequence, under high power decoupling from ^1^H nuclei, using a recycle delay of 120 s, and accumulating 1320 transients. All the experiments were carried out under magic angle spinning (MAS), with a frequency of 12 kHz, using air as spinning gas, and at a temperature of 20 °C. ^31^P chemical shift scale was referred to the signal of H_3_PO_4_ (85%) at 0 ppm.

Infrared spectra were recorded with a Spectrum Two Fourier Transform Infrared Spectrometer (PerkinElmer, Waltham, MA, USA) over the wavenumber range of 400–4000 cm^−1^ with a resolution of 4 cm^−1^ using 16 scansions. The spectra were registered on thin films of polymer samples obtained by solution casting on a KBr window of chloroform solutions prepared by dissolving 5 mg of each sample in 1 mL of chloroform. The composition of PMMA-*b*-PS and PMMA-*b*-PS/fl-bP was accomplished by using a calibration curve, which was prepared by acquiring FT-IR spectra of PS/PMMA blends of known compositions. The FT-IR spectra of PS/PMMA blends show two well-resolved bands at 1730 and 698 cm^−1^ due to the C=O stretching of the ester group of PMMA and C-C bending out of the plane of PS, respectively. The ratio between the area of these bands was reported versus the molar composition.

## 3. Results

### 3.1. PS/PMMA/fl-bP Composites by Solution Blending

Three PS/PMMA/fl-bP composites were prepared by keeping the PS/PMMA weight ratio constant (50/50 wt.%) and adding three different fl-bP suspensions, respectively. Each of the three suspensions contains flakes with different average hydrodynamic diameters: fl-bP0 (CHP suspension after exfoliation), fl-bP3000, and fl-bP500, which were selected by centrifugation. The amount of fl-bP in PS/PMMA/fl-bP0 was 1 wt.%, whereas in PS/PMMA/fl-bP3000 and PS/PMMA/fl-bP5000, it was estimated to be about 1 wt.% and 0.6 wt.%, respectively.

**Figure 2:**
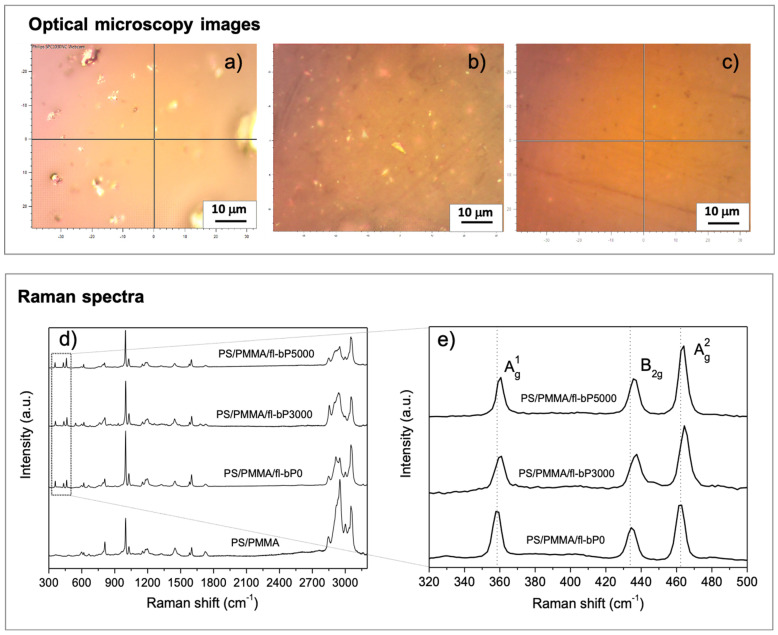
Representative optical microscopy images of (**a**) PS/PMMA/fl-bP0, (**b**) PS/PMMA/fl-bP3000, and (**c**) PS/PMMA/fl-bP5000. Representative Raman spectra (*λ* = 532 nm) of PS/PMMA and PS/PMMA/fl-bP composites (**d**) and blow up of the Raman spectra of PS/PMMA/fl-bP composites in the 320–500 cm^−1^ range (**e**).

The PS/PMMA/fl-bP composites were characterized by micro-Raman spectroscopy. The optical microscopy images show bright inclusions of micrometric size consisting of fl-bP, as reported previously (Figure 2) [39]. This result shows that fl-bP is present in the form of flake aggregates, although the presence of single flakes cannot be excluded by this test. Dispersion and spatial distribution of inclusions within composites depend on the type of fl-bP suspension and its concentration. The optical image of PS/PMMA/fl-bP0 (Figure 2a) shows large aggregates (1–5 μm) due to the large size of fl-bP flakes in the fl-bP0 suspension (the hydrodynamic diameter is ca. 360 nm and non-exfoliated bP is likely present, as reported in the Materials and Methods section) as well as possible aggregation between the flakes. The optical image of PS/PMMA/fl-bP3000 reveals a finer distribution of fl-bP flakes (below 1 μm) and the presence of only a few aggregates due to the smaller hydrodynamic diameter of the fl-bP3000 suspension (Figure 2b). Additionally, no evidence of aggregates can be observed in the optical microscopy image of PS/PMMA/bP5000 (Figure 2c), even though the Raman spectrum confirms the presence of fl-bP. Notably, Raman spectra of the three composites show vibrational modes of both PS and PMMA [PS: 620 cm^−1^ (ring deformation mode), 999 cm^−1^ (ring breathing mode), 1197 cm^−1^, 1583 cm^−1^ (C=C stretch), 1600 cm^−1^ (ring-skeletal stretch), 2908 cm^−1^ (tertiary CH stretching), 3052 cm^−1^ (aromatic CH stretching); PMMA: 599 cm^−1^, 810 cm^−1^, 1448 cm^−1^ (CH_2_ deformation), 1726 cm^−1^ (C=O stretching), 2851 cm^−1^ (CH_2_ symmetric stretching), 2951 (CH_3_ symmetric stretching) cm^−1^], and also three distinct bands at about 360, 430, and 460 cm^−1^ corresponding to the out-of-plane A^1^_g_ phonon mode, to the in-plane modes along the zig-zag direction B_2g_, and along the armchair direction A^2^_g_ of bP (Figure 2d) [50]. 

The retention of bP vibrational modes in the Raman spectra suggests that fl-bP was not degraded. Additionally, the blow-up of the 320–500 cm^−1^ range (Figure 2e) indicates that the three Raman bands of bP shifted to the blue from PS/PMMA/fl-bP0 to PS/PMMA/fl-bP3000 and PS/PMMA/fl-bP5000. This result is consistent with a decrease in the hydrodynamic diameter from the fl-bP0 to the fl-bP5000 suspension and with a corresponding lower average number of layers of fl-bP flakes [19]. Raman spectra collected in different discrete locations, including bright inclusions and portions of the samples apparently bP-free, are provided in the Supplementary Materials Figure S1). The Raman spectrum of bright inclusions confirmed that it consists of bP showing intense signals of Raman active modes of bP and weak vibrational modes due to the polymeric phase, as in the case of PS/PMMA/fl-bP3000 (Figure S1a). Additionally, spectra collected in areas apparently bP-free exhibited vibrational modes of both fl-bP and polymers. In the case of the sample PS/PMMA/bP5000, bright inclusions with smaller dimensions were observed, but it was not possible to limit the acquisition of the Raman spectrum only to their area. However, by comparing spectra acquired at different locations, the characteristic vibrational modes of bP and PMMA/PS blend can be observed in all the analyzed areas (Figure S1b). Therefore, the presence of the three bP peaks also in regions that are apparently bP-free indicates good dispersion of fl-bP, especially in the case of the sample PS/PMMA/fl-bP5000 for which this condition is satisfied in the whole area [39].

**Figure 3:**
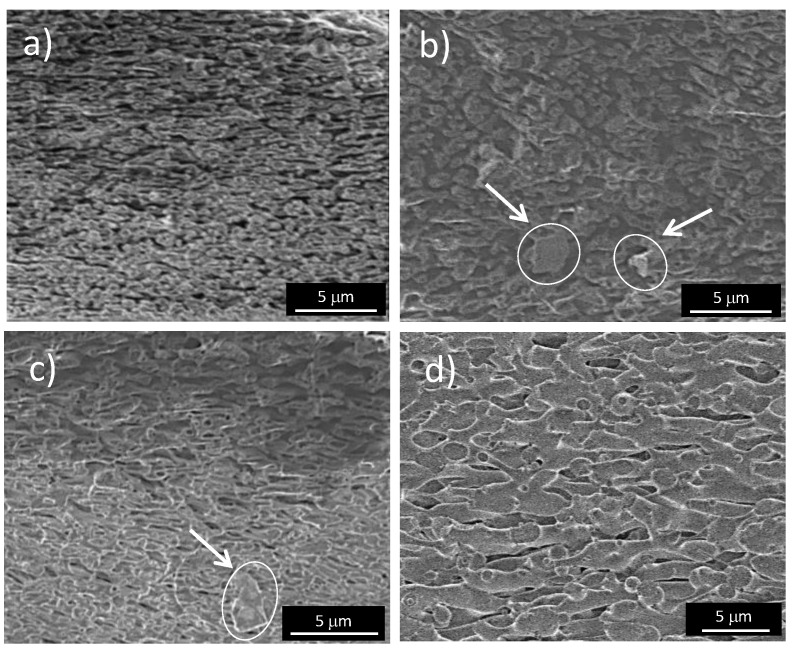
SEM images of selectively extracted PS/PMMA (**a**), PS/PMMA/fl-bP0 (**b**), PS/PMMA/fl-bP3000 (**c**), and PS/PMMA/fl-bP5000 (**d**). The black domains represent the cyclohexane-etched PS phase. The arrows in the images (**b**,**c**) indicate the fl-bP.

The morphology of the polymer phase was investigated using SEM to determine whether the fl-bP caused a variation compared to the PS/PMMA blend. SEM micrographs were registered on cryofractured and PS etched samples. The PMMA/PS blend (Figure 3a) shows a continuous structure of the PMMA phase and elongated PS cavities inter-connected, suggesting a co-continuous or quasi co-continuous microstructure, as previously reported [51]. The addition of fl-bP coarsened the microstructure. Micrographs of composites show a slight increase in the size of PS domains compared with the PS/PMMA blend (Figure 3b,c), and the effect was more evident using fl-bP5000 suspension with a lower hydrodynamic diameter of the flakes. No evidence of fl-bP was observed in SEM micrographs of composites collected before etching (Figure S2, Supplementary Materials), while micrographs of fractured and etched PS/PMMA/fl-bP0 and PS/PMMA/fl-bP3000 confirmed the presence of micrometric fl-bP aggregates previously observed by optical microscopy (Figure 3b,c). However, no fl-bP, as aggregates or single flakes, were detected from the micrograph of etched PS/PMMA/fl-bP5000 (Figure 3d), which is likely due to the small size of flakes contained in the fl-bP5000 suspension and to the lower concentration of fl-bP in this composite (0.6 wt.%).

**Figure 4:**
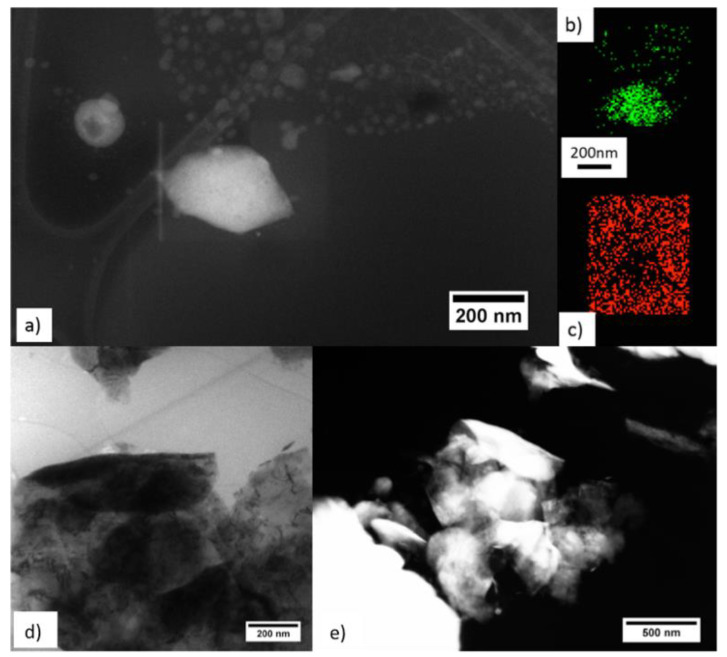
Sample PS/PMMA/fl-bP5000: STEM image of a squared fl-bP (**a**), EDX map of the fl-bP sheet (**b**), in green the P map, and in red the C map of the area that surrounds the fl-bP sheet (**c**). Conventional TEM (**d**) and STEM (**e**) images of a large agglomerate of fl-bP.

The presence of fl-bP in the PS/PMMA/fl-bP5000 sample was verified by STEM and TEM analysis. Micrographs of the sample showed typical squared fl-bP flakes of about 200 nm (Figure 4a), connected or aggregated (Figure 4d,e), and several smaller flakes (average size less than 50 nm). These nanostructures are fl-bP flakes surrounded by carbon, as evidenced by EDX elemental maps (Figure 4b,c). The flakes, with a size distribution ranging between 10 and 400 nm, appeared uniformly distributed in the polymer matrix (Figure S3, Supplementary Materials), although it was not possible to distinguish the two polymeric phases. The presence of small fl-bP flakes could be due to the prolonged sonication of bP used for fl-bP preparation. During sonication, cavitation can break the larger sheets, thus forming small fragments that coexist with the larger flakes [52,53,54].

**Figure 5:**
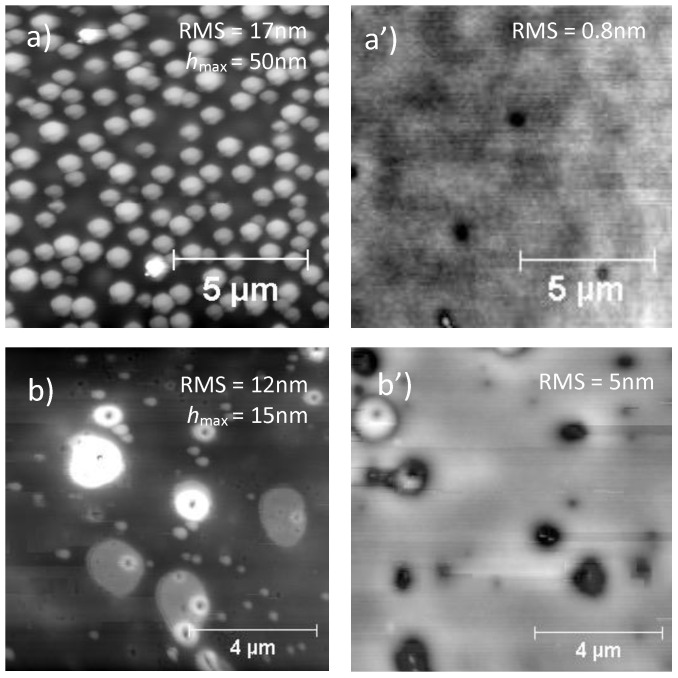
AFM topography images of PS/PMMA blend and PS/PMMA/fl-bP0 before and after annealing: (**a**) PS/PMMA, 1% wt./vol toluene (thin film), spin-cast, and (**a’**) after annealing; (**b**) PS/PMMA/fl-bP0, 1% wt./vol toluene, spin-cast, and (**b’**) after annealing. The RMS roughness and maximum height of bumps (*h*_max_) are reported.

As the principal application of these materials is as ultrathin films for devices and membranes [39,40], AFM analysis was used to investigate the surface morphology (topography). AFM images of PS/PMMA blend and PS/PMMA/fl-bP composites were collected on spin-cast film before and after the annealing process (150 °C in a vacuum oven for 24 h). Before annealing, the AFM image of the PS/PMMA blend showed a granular surface with bumps (Figure 5a). 

To demonstrate the nature of the bumps, films of PS/PMMA on silicon wafers were immersed in cyclohexane and acetic acid to remove PS and PMMA, respectively. The AFM images after the etching processes revealed the residual PMMA phase in one case and the residual PS phase in the other (Figure S4, Supplementary Materials). After dissolving PS in cyclohexane, the topography still showed a granular structure with increased height of the bumps, while after dissolving PMMA in acetic acid, the film was detached from the wafer surface and its topography appeared pitted with PS as the continuous phase. This result, in agreement with the literature, suggests that bumps are made of PMMA [55,56,57].

Upon annealing above the glass transition temperatures of the two polymers, the PS/PMMA surface appeared relatively flat (as confirmed by low RMS value) with a few pits (Figure 5a’). This effect is due to the surface segregation of PS by dewetting that minimizes the polymer–air interfacial free energy giving a relatively flat surface [58]. The PS/PMMA/fl-bP0 showed a topography with bumps of irregular shape and dimensions from less than 1 micron to several microns (Figure 5b). In addition, the image showed small structures segregated in the PMMA phases, which are likely fl-bP sheets or fl-bP agglomerates. However, upon annealing, these fl-bP sheets/agglomerates were not more visible probably because they were submerged by the dewetted PS phase and segregated into the PMMA phase (Figure 5b’). This result may indicate a more favorable interaction of fl-bP with PMMA compared to PS. 

**Figure 6:**
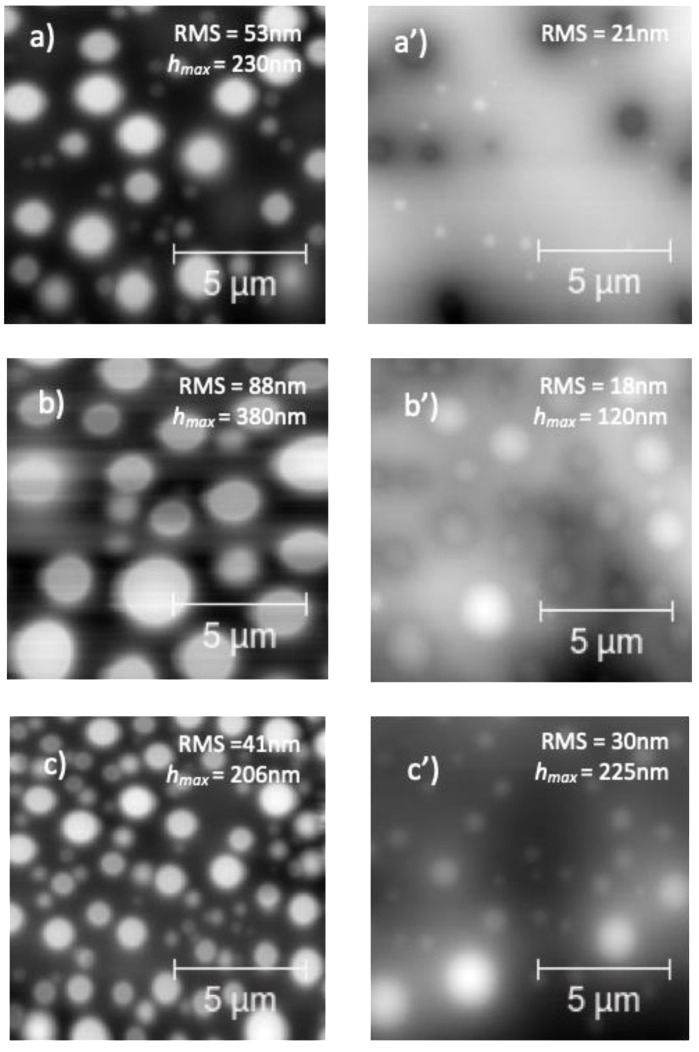
AFM topography of PS/PMMA (**a**,**a’**), PS/PMMA/fl-bP3000 (**b**,**b’**), and PS/PMMA/fl-bP5000 (**c**,**c’**), 5% wt./vol toluene, as spin-cast (**a**–**c**), and upon annealing (**a’**–**c’**). The RMS roughness and the maximum height of the bumps (*h*_max_) are reported.

The topography of PS/PMMA/fl-bP3000 and PS/PMMA/fl-bP5000 films (Figure 6) was similar to that of the unfilled blend: the bumps were regular and homogeneously distributed. However, the average diameter of the bumps in the PS/PMMA/fl-bP3000 sample was higher than that of the PS/PMMA blend, and the number of domains was lower (Figure 6b); the roughness was also higher than that of the blend, as well as the height of the bumps. Conversely, the addition of fl-bP5000 suspension, with the lowest average hydrodynamic diameter of fl-bP flakes (ca. 212 nm), reduced the diameter of the bumps and increased their number within the measured area (Figure 6c); in addition, the roughness was lower compared to the blend as well as the height of the bumps. A possible reason for the observed behavior may be a change in the rheological behavior of PMMA, where the fl-bP flakes were likely preferentially located with an increase in the viscosity of the PMMA phase that causes the decrease in the diameter of PMMA domains [59]. In particular, the effect is more evident for the smaller fl-bP flakes that better match the size of the polymer phase. Interestingly, after the annealing process, the topography of both composites remained granular, suggesting that the PS dewetting was delayed by fl-bP (Figure 6a’–c’). However, fl-bP was not directly visible on the surface of the films, either before or after the annealing. 

**Figure 7:**
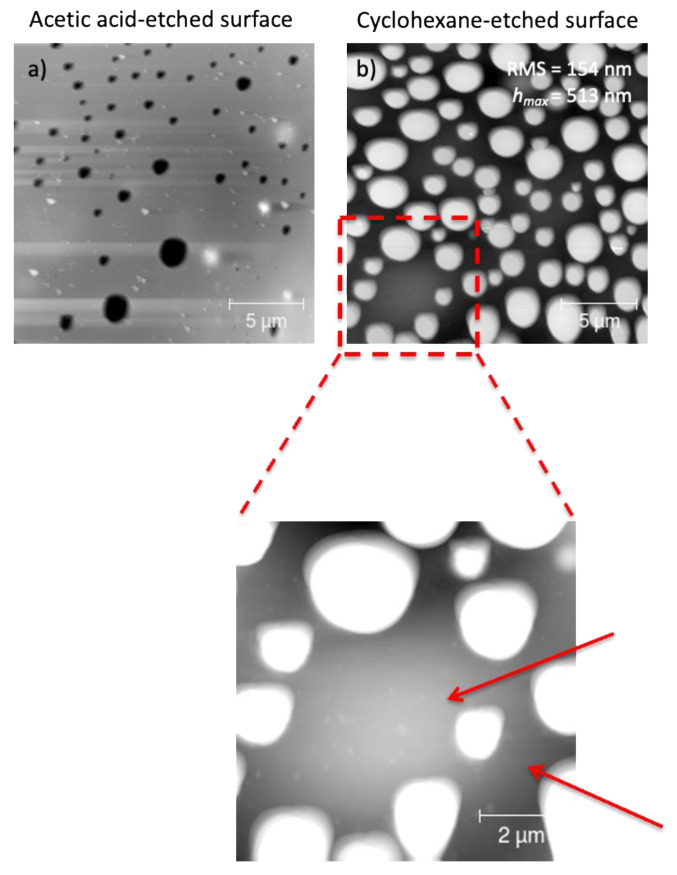
AFM topography of annealed PS/PMMA/fl-bP3000 films after acetic acid (**a**) and cyclohexane (**b**) etching, respectively. Films were prepared from 5% wt./vol toluene solution. The inset shows the blow-up of the cyclohexane-etched film, and the arrows indicate the fl-bP flakes.

**Figure 8:**
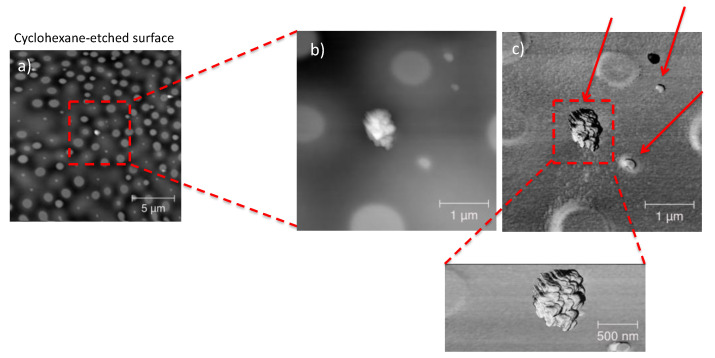
AFM topography of a thin film of PS/PMMA/fl-bP5000 after cyclohexane etching (**a**). The film was prepared from 1% wt./vol toluene solution. The inset shows a blow-up (**b**) and the blow-up in elastic contrast (**c**). The arrows indicate the fl-bP.

To check the distribution of fl-bP within the PS/PMMA matrix in both PS/PMMA/fl-bP3000 and PS/PMMA/fl-bP5000, AFM images were acquired after cyclohexane and acid acetic etching (Figure 7 and Figure 8). Notably, after cyclohexane etching of the PS/PMMA/fl-bP3000 film, structures of 100–200 nm of diameter and 5–20 nm thickness appeared at the bottom of the bumps. These structures are made of fl-bP, as confirmed by micro-Raman spectroscopy. On the contrary, no evidence of fl-bP was observed on the acetic acid-etched surface, which is consistent with the preferential localization of fl-bP flakes into the PMMA phase.

**Figure 9:**
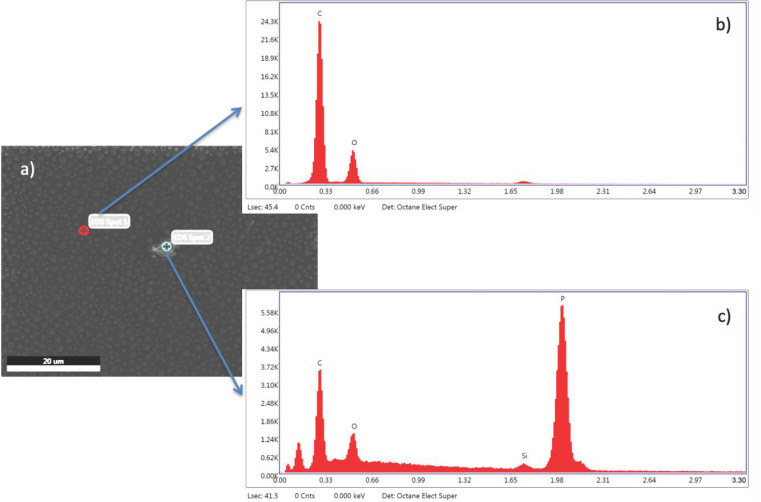
SEM images of PS/PMMA/fl-bP5000 thin film on a silicon wafer after cyclohexane etching (**a**). EDXS spectra collected on a bP-free area (**b**) and on an fl-bP agglomerate (**c**).

Similar results were achieved for the PS/PMMA/fl-bP5000 composite (Figure 8). In this case, the AFM topography in elastic contrast showed small flakes (*h* = 20–50 nm) dispersed in the PMMA phase after PS etching and an agglomerate of stacked bP layers (*h* = 150 nm), as confirmed by EDX-SEM analysis (Figure 9). Agglomerates may have formed due to the etching process. The cyclohexane decreases the PS viscosity that becomes a convenient medium of diffusion of the flakes. In this condition, fl-bP can migrate and may be subjected to coarsening and coalescence.

**Figure 10:**
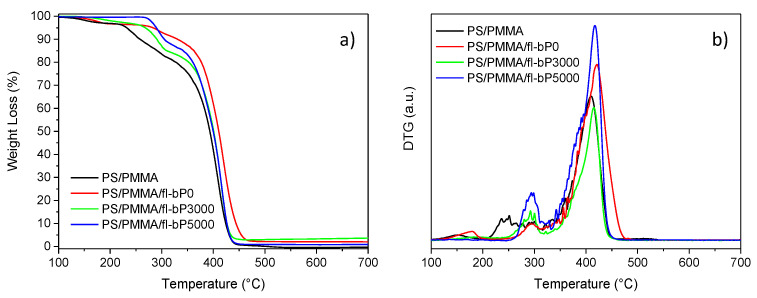
TG (**a**) and DTG (**b**) curves of PS/PMMA, PS/PMMA/fl-bP0, PS/PMMA/fl-bP3000, and PS/PMMA/fl-bP5000 carried out under nitrogen flow.

The thermal stability of PS, PMMA, PS/PMMA blend (50/50 wt.%), and PS/PMMA/fl-bP composites was evaluated by TGA. Thermogravimetric (TG) and derivative thermogravimetric (DTG) curves are shown in Figure 10 and Figure S5 (Supplementary Materials), and data are reported in Table 2. PS degraded in a single step with an onset temperature of 394 °C and a maximum degradation rate of 414 °C. The thermogravimetric curve of PMMA revealed two main steps of weight loss with a maximum degradation rate at 282 and 389 °C (random scission initiation), respectively, as reported previously [60]. The decomposition of PS/PMMA occurred in two steps in the temperature range of 300–500 °C.

The first step can be attributed to the degradation of PMMA and the second to a combination of PS and PMMA degradation. Similarly, TG/DTG curves of PS/PMMA/fl-bP composites showed two main steps of degradation that can be explained as for the blend. Remarkably, the fl-bP degradation occurs between 410 and 480 °C, overlapped with the second degradation step of the polymers [39,61]. Interestingly, the initial decomposition temperature of the composites increased significantly compared to the blend (about 40–50 °C) indicating higher thermal stability [26]. Composites also showed an increment of the temperature corresponding to the maximum degradation rate (Table 2). These encouraging results about the improved thermal stability of the PS/PMMA blend agree with previous data indicating that fl-bP inhibits mass transfer and provides thermal insulation to shield the underlying polymer from the heat source [26,28,29,30,31,32,33,34,35,36,37]. Similar results have also been reported for other types of ultrathin 2D nanomaterials dispersed both in thermoplastic and thermosetting polymers [62,63,64].

Furthermore, to verify possible interactions between PS, PMMA, and fl-bP, and to investigate how the PS/PMMA miscibility is affected by fl-bP, the glass transition temperature (*T*_g_) of composites was measured by DSC and compared with that of the neat blend and pure components (Table 2). The DSC thermogram of PS/PMMA blend showed two separated glass transitions corresponding to PS and PMMA phases, respectively, indicating the non-miscibility of the blend. This condition was maintained even after the fl-bP addition, since the thermograms of PS/PMMA/fl-bP0, PS/PMMA/fl-bP3000, and PS/PMMA/fl-bP5000 composites still showed two distinguishable *T*_g_ (Figure S6, Supplementary Materials). These results are consistent with SEM morphology showing polymer phase separation for the two composites as well. However, glass transition temperatures were higher for the composites than for the pure blend, suggesting an additional separation effect of the two phases induced by the presence of fl-bP.

### 3.2. PMMA-b-PS/fl-bP Composite via In Situ RAFT Polymerization

**Figure 11:**
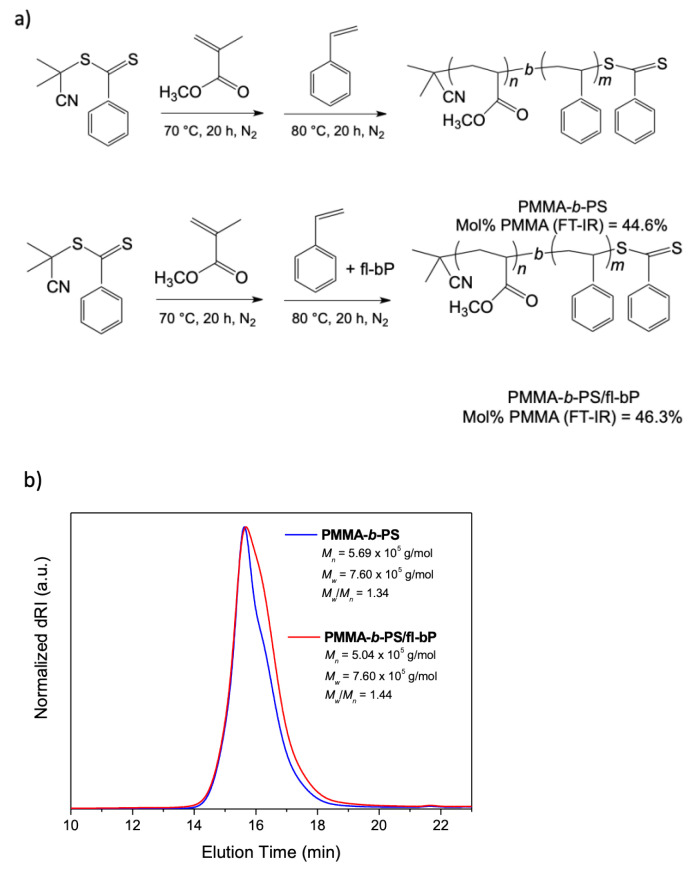
Synthesis of the diblock copolymer PMMA-*b*-PS and of the composite PMMA-*b*-PS/fl-bP by RAFT polymerization: (**a**) general reaction scheme; (**b**) SEC traces of PMMA-*b*-PS and PMMA-*b*-PS/fl-bP.

To probe the possibility of embedding fl-bP into a block copolymer, as a method of encapsulation allowing us to obtain a wide range of hybrid structures with possible confined geometry, a PMMA-*b*-PS/fl-bP composite was prepared by in situ RAFT polymerization carried out in bulk after LPE of bP into styrene (Figure 11a). First, we prepared a macro-RAFT agent by in-bulk RAFT polymerization of MMA using a dithiobenzoate RAFT agent (CPDB) with a predetermined degree of polymerization targeted through the monomer to RAFT agent ratio, as reported by Jana et al. [49]. Then, a PMMA-*b*-PS copolymer, as a blank, was prepared by reaction between the macro-RAFT and styrene. A quite narrow dispersed PMMA was synthesized, and when the PMMA chain was extended with the polymerization of styrene to yield the di-block copolymer, while styrene conversion was relatively low, quite good control of molecular weight and dispersity was maintained (Table 1, Figure 11b).

When the styrene suspension containing fl-bP and prepared by LPE was used to synthesize the PS block from the macro-RAFT, the styrene conversion was further reduced compared to the diblock copolymer, but molecular weight distribution was enough controlled. A PMMA-*b*-PS/fl-bP composite containing 1.2 wt.% of fl-bP was obtained. Notably, for both PMMA-*b*-PS and PMMA-*b*-PS/fl-bP samples, the molecular weight of the PS block was lower than expected, and a relatively high dispersity was found. Probably, the high viscosity generated in the bulk systems plays a critical role in polymerization control, limiting the diffusion of the molecules, giving lower mobility to radicals, and favoring termination reactions. Additionally, fl-bP may interfere with the mechanism of transfer of the RAFT agent also determining chain end by radical coupling reaction [65,66].

**Figure 12:**
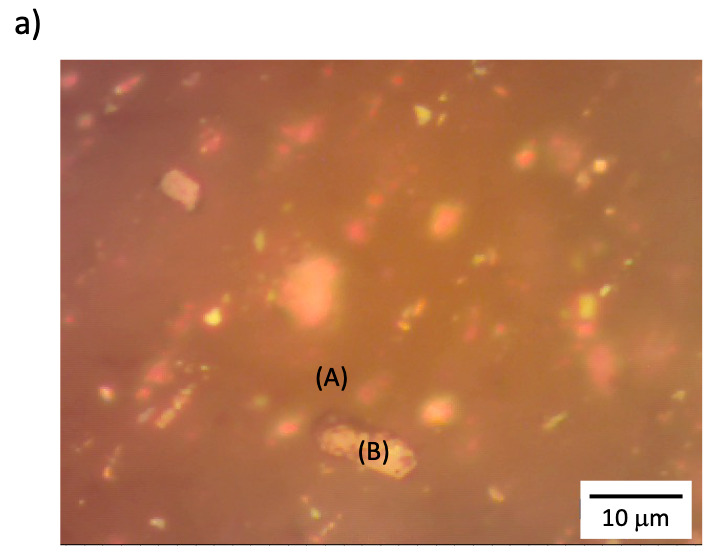

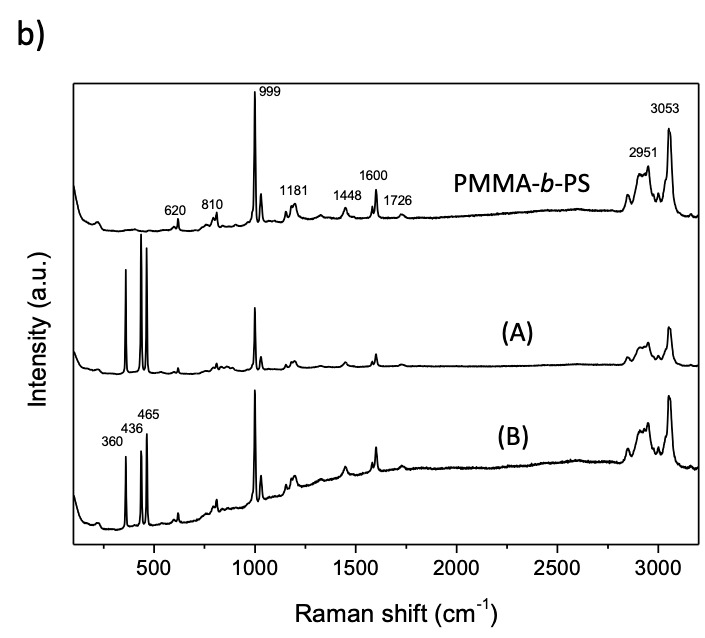
Representative optical microscopy of PMMA-*b*-PS/fl-bP (**a**) and Raman spectra recorded in two different points (A,B) and compared with the Raman spectrum of the diblock copolymer PMMA-*b*-PS (**b**).

Optical microscopy images of PMMA-*b*-PS/fl-bP showed a wide range of size distribution of fl-bP into the copolymer. Big agglomerates of a few microns can be observed (Figure 12a), and the corresponding Raman spectrum (Figure 12b, spectrum B) confirms that they consist of bP. 

As previously reported for PS/PMMA/fl-bP samples, the Raman spectrum collected in an apparently bP-free area showed all the characteristic vibrational modes of the copolymer and the three distinct bands corresponding to A^1^_g_, B_2g_, and A^2^_g_ modes of bP (Figure 12b, spectrum A). This result likely indicates that smaller fl-bP flakes not visible with the optical microscopy are also dispersed into the matrix. However, while the AFM topography of the copolymer showed an irregular wormlike network structure with a roughness of 0.7 nm, the AFM image of PMMA-*b*-PS/fl-bP is dominated by brighter regions with a height of about 500 nm that are the aggregates of fl-bP (Figure S7, Supplementary Materials). Hence, the surface roughness was not uniform throughout the entire film, confirming that the size of fl-bP differed and it was not possible to recognize a preferential localization in one of the two polymeric phases.

**Figure 13:**
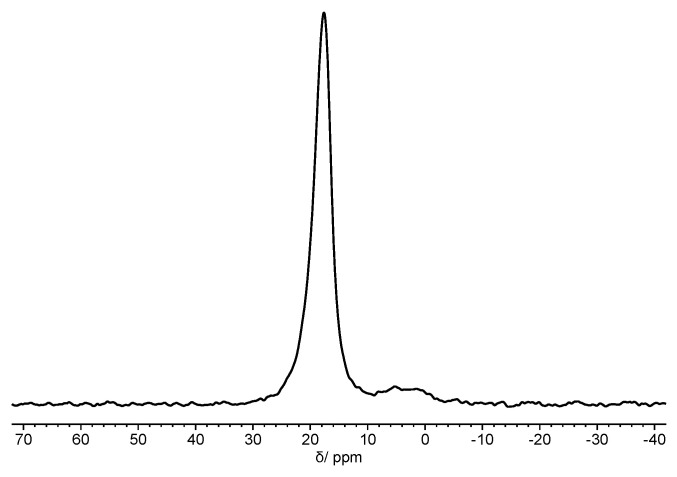
^31^P SSNMR spectrum of PMMA-*b*-PS/fl-bP.

Interestingly, in agreement with previous studies [39], this sample confirmed that the in situ polymerization is very powerful in avoiding the oxidation of bP. Indeed, ^31^P SSNMR spectrum of PMMA-*b*-PS/fl-bP shows a signal at 18 ppm, typical of fl-bP (Figure 13). Weak broad signals are also observable between 0 and 10 ppm, which can be ascribed to products of degradative oxidation of fl-bP, including different phosphorus oxyacid anions such as HPO_3_^2−^ and PO_4_^3−^. From ^31^P-signal integration, it is possible to estimate that P atoms of the oxidation products account for only 6% of the whole amount of P atoms, indicating a very good control of the bP preservation from oxidation during all preparation steps and confirming that the in situ polymerization method is very successful in preserving the bP structure. 

In addition, TGA showed an increment of the thermal stability of PMMA-*b*-PS/fl-bP compared to PMMA-*b*-PS with an increase in *T*_mdr_ (Figure S8, Supplementary Materials). The TGA residue of the composite was higher than that of the block copolymer (2.90 and 3.75 wt.% at 550 °C, respectively) suggesting that fl-bP flakes contributed to the char residue. However, considering the bP degradation between 400 and 550 °C [39,67], it was not possible to estimate the amount of fl-bP from the TGA residue. 

## 4. Conclusions

In conclusion, this study describes the first example of fl-bP inclusion into a biphasic polymer blend made of PS/PMMA (50/50 wt.%) and into a block PMMA-*b*-PS copolymer. First, bulk bP was exfoliated by LPE in CHP using a probe sonicator, and the suspension was used as it is or after centrifugation at a different speed for selecting fl-bP populations with a relatively low and narrow average hydrodynamic size distribution. Second, PS/PMMA/fl-bP composites were prepared by solution blending. Micro-Raman spectroscopy demonstrated that fl-bP remained crystalline and not oxidized, thus preserving its potential outstanding properties. In addition, fl-bP was dispersed and distributed into the polymer blend based on its size and concentration. SEM analysis of PS/PMMA/fl-bP composites showed that fl-bP suppressed the coalescence of PS and PMMA phases revealing the localization of the flakes at the interface. TEM and STEM micrographs of PS/PMMA/fl-bP5000 prepared with the smallest fl-bP flakes showed typical squared fl-bP flakes of about 200 nm, connected or aggregated. This analysis revealed the presence of several smaller flakes of spherical shape with an average size less than 50 nm. Furthermore, AFM analysis showed that fl-bP incorporation into PS/PMMA determined a change in topography of the blend by delaying the PS dewetting. In addition, the selective etching of PS generated the isolation of PMMA bumps and the appearance of fl-bP flakes at the basis of the bumps. This evidence revealed the possible generation of percolation patterns. PS/PMMA/fl-bP composites also exhibited improved thermal stability with decreasing average hydrodynamic size of fl-bP flakes, thus confirming that fl-bP successfully inhibits the mass transfer and provides thermal insulation. 

Finally, a block copolymer/fl-bP composite (PMMA-*b*-PS/fl-bP) was prepared via in situ reversible RAFT polymerization with direct sonication-assisted exfoliation of bP into styrene. This sample showed an excellent preservation of bP structure as confirmed by ^31^P SSNMR and Raman spectroscopy, even though the fl-bP dispersion was limited by the heterogeneous size of fl-bP. The versatility of this approach may be exploited in future synthesis of amphiphilic block copolymers containing small-size fl-bP flakes, which may be assembled into different morphologies. In the case of both binary polymer blend composites and block copolymer composites, future efforts might usefully focus on the properties of devices made of these materials. 

## Figures and Tables

**Table 1:** Polymerizations: monomer conversion and molecular weight.

Sample	Conversion ^1^ (%)	*M_n_* (g/mol)	*M_w_/M_n_*
PMMA-CPDB	67	29,700	1.25
PMMA-*b*-PS	67 + 34	56,900	1.34
PMMA-*b*-PS/fl-bP	67 + 24	50,400	1.44

^1^ Monomer conversion was determined by weight method.

**Table 2:** DSC and TGA results: glass transition temperature (*T*_g_), onset temperature of decomposition (*T*_onset_), and temperature corresponding to the maximum decomposition rate (*T*_mdr_).

Sample	*T*_g,PS_^1^ (°C)	*T*_g,PMMA_^1^ (°C)	*T*_onset_^2^ (°C)	*T*_mdr_^3^ (°C)
PS	105.3	−	394	414
PMMA	−	122.4	261	282; 389
PS/PMMA	103.6	119.8	223	251; 411
PS/PMMA/fl-bP0	105.0	125.0	275	294; 422
PS/PMMA/fl-bP3000	107.0	124.5	264	292; 415
PS/PMMA/fl-bP5000	104.4	122.0	274	295; 417

^1^*T*_g_ was calculated from the second heating scan. ^2^ The onset temperature was calculated from the degradation step above 200 °C as the intercept of tangents before and after the degradation step. The weight loss before 200 °C was attributed to loss of the trapped solvent. ^3^ The temperature corresponding to the maximum decomposition rate was determined from DTG curves as the maximum of the peak.

## Data Availability

The data presented in this study are available on request from the authors.

## Supplementary Materials

The following are available online at https://www.mdpi.com/article/10.3390/nano11081996/s1, Table S1: List of all the samples prepared: sample name, method of preparation, and fl-bP concentration; Figure S1: Representative optical microscopy images of PS/PMMA/fl-bP3000 and PS/PMMA/fl-bP5000 (a,b, left side). Representative Raman spectra of PS/PMMA/fl-bP3000 and PS/PMMA/fl-bP5000 (a,b, right side) recorded in different areas and compared with the Raman spectrum of PS/PMMA blend; Figure S2: Representative SEM micrographs of PS/PMMA blend (a,b), PS/PMMA/fl-bP3000 (c,d), and PS/PMMA/fl-bP5000 (e,f); Figure S3: (a) STEM image of the sample PS/PMMA/fl-bP5000: the green rectangular highlights the area where the EDX elemental map was collected; (b) phosphorus STEM-EDX map; (c) EDX spectrum corresponding to the map reported in b; Figure S4: AFM topography of PS/PMMA blend, 5% wt./vol toluene, spin-cast film (a), acetic acid-etched film (b), and cyclohexane-etched film (c). Schemes illustrating the profile of surface topography are reported in the three cases; Figure S5: TG and DTG curves of PS and PMMA under nitrogen flow; Figure S6: DSC curves second heating scan of PS (a), PS/PMMA (b), PS/PMMA/fl-bP0 (c), PS/PMMA/fl-bP3000 (d), PS/PMMA/fl-bP5000 (e), and PMMA (f); Figure S7: AFM topography of PMMA-*b*-PS (a) and PMMA-*b*-PS/fl-bP (b) 5% wt./vol toluene, as spin-cast; Figure S8: TG and DTG curves of PMMA-*b*-PS and PMMA-*b*-PS/fl-bP under nitrogen flow.
