# Supplementary material for: Dispersion of Few-Layer Black Phosphorus in Binary Polymer Blend and Block Copolymer Matrices
Source: Nanomaterials (Basel). 2021 Aug 3;11(8):1996. doi: 10.3390/nano11081996 (PMC8398111; doi:10.3390/nano11081996)
Supplement: Supplementary file 1 [file nanomaterials-11-01996-s001.zip › nanomaterials-1270897-supplementary.pdf]

nanomaterials

MDPI

Supplementary Materials

Dispersion of Few-Layer Black Phosphorus in Binary Polymer Blend and Block Copolymer Matrices

Serena Coiai [1,*], Elisa Passaglia [1], Simone Pinna [1], Stefano Legnaioli [1], Silvia Borsacchi [1,2], Franco Dinelli [3], Anna Maria Ferretti [4], Maria Caporali [5], Manuel Serrano-Ruiz [5], Maurizio Peruzzini [5] and Francesca Cicogna [1]

1 CNR-ICCOM, Consiglio Nazionale delle Ricerche, Istituto di Chimica dei Composti OrganoMetallici, SS Pisa, Via Moruzzi 1, 56124 Pisa, Italy; elisa.passaglia@pi.iccom.cnr.it (E.P.); simop93@hotmail.it (S.P.); stefano.legnaioli@pi.iccom.cnr.it (S.L.); silvia.borsacchi@pi.iccom.cnr.it (S.B.); francesca.cicogna@pi.iccom.cnr.it (F.C.)
2 CISUP, Centro per l'Integrazione della Strumentazione Scientifica dell'Università di Pisa, 56126 Pisa, Italy
3 CNR-INO, Consiglio Nazionale delle Ricerche, Istituto Nazionale di Ottica, Via Moruzzi 1, 56124 Pisa, Italy; franco.dinelli@ino.cnr.it
4 CNR-SCITEC, Consiglio Nazionale delle Ricerche, Istituto di Scienze e Tecnologie Chimiche, Via G. Fantoli 16/15, 20138 Milano, Italy; anna.ferretti@scitec.cnr.it
5 CNR-ICCOM, Consiglio Nazionale delle Ricerche, Istituto di Chimica dei Composti OrganoMetallici, Via Madonna del Piano 10, 50019 Sesto Fiorentino (FI), Italy; maria.caporali@iccom.cnr.it (M.C.); manuel.serrano@iccom.cnr.it (M.S.-R.); maurizio.peruzzini@iccom.cnr.it (M.P.)
* Correspondence: serena.coiai@pi.iccom.cnr.it, Tel.: +39-050-315-2556

Table S1. List of all the samples prepared: sample name, method of preparation, and fl-bP concentration.

Sample	Method of Preparation	fl-bP Concentration
suspension fl-bP0 [1]	LPE in CHP	1 mg/ml
suspension fl-bP3000 [1]	LPE in CHP + centrifugation	0.5 mg/ml
suspension fl-bP5000 [1]	LPE in CHP+ centrifugation	0.3 mg/ml
PS/PMMA [2]	Solution blending	n.p. [3]
PS/PMMA/fl-bP0 [2]	Solution blending	1 wt%
PS/PMMA/fl-bP3000 [2]	Solution blending	1 wt%
PS/PMMA/fl-bP5000 [2]	Solution blending	0.6 wt%
PMMA-CPDB	RAFT polymerization	n.p. [3]
PMMA-*b*-PS	RAFT polymerization	n.p. [3]
PMMA-*b*-PS/fl-bP	LPE in styrene + RAFT polymerization	1.2 wt%

[1] The suspension fl-bP0 was obtained after LPE. Suspension fl-bP3000 and suspension fl-bP5000 were obtained after LPE followed by centrifugation at 3000 and 5000 rpm, respectively. [2] The PS/PMMA ratio was 50/50 wt.%. [3] n.p.: non-present.

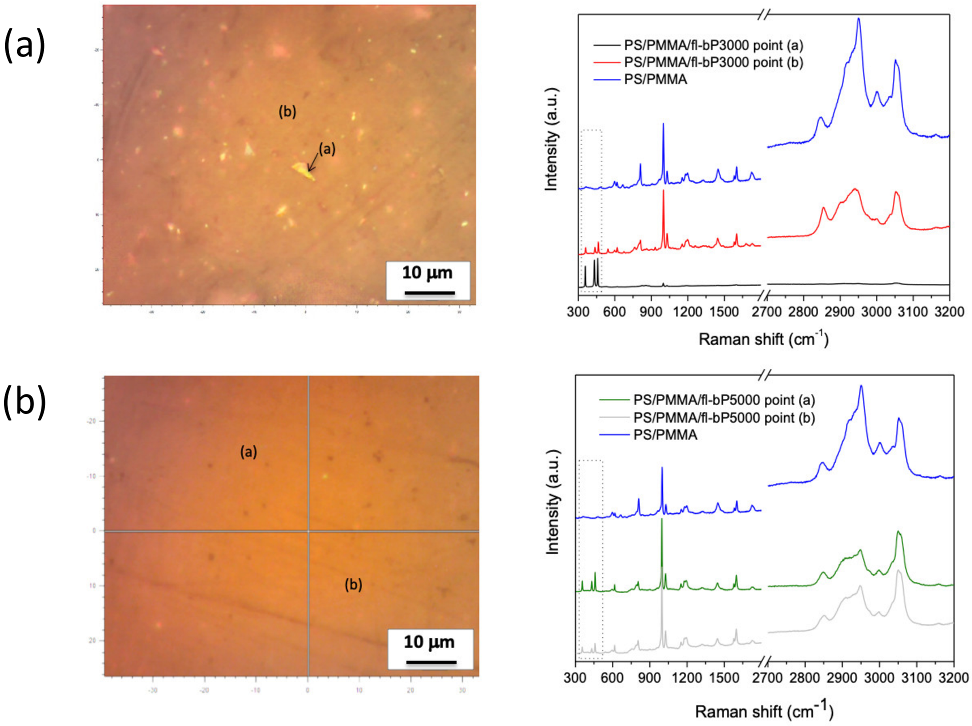

Figure S1. Representative optical microscopy images of PS/PMMA/fl-bP3000 and PS/PMMA/fl-bP5000 (**a** and **b**, left side). Representative Raman spectra of PS/PMMA/fl-bP3000 and PS/PMMA/fl-bP5000 (**a** and **b**, right side) recorded in different areas and compared with the Raman spectrum of PS/PMMA blend.

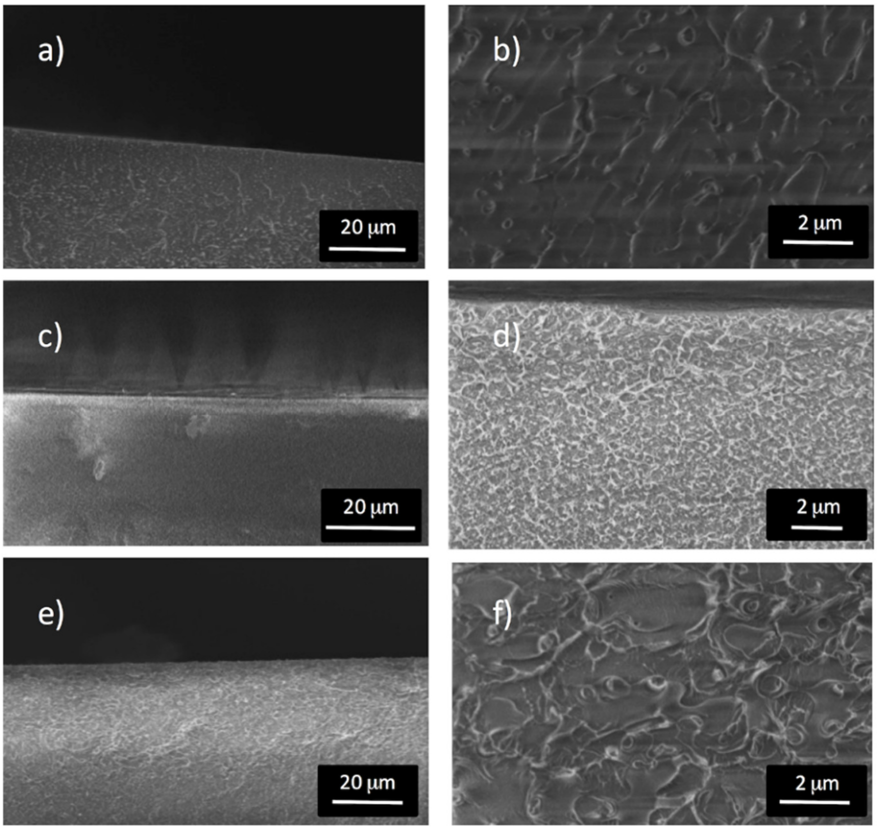

Figure S2. Representative SEM micrographs of PS/PMMA blend (**a**,**b**), PS/PMMA/fl-bP3000 (**c**,**d**), and PS/PMMA/fl-bP5000 (**e**,**f**).

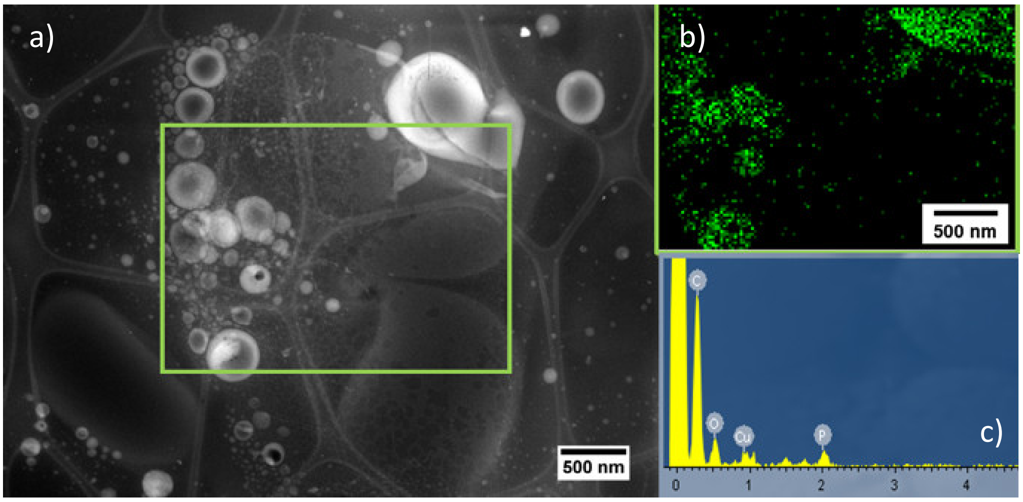

Figure S3. (**a**) STEM image of the sample PS/PMMA/fl-bP5000: the green rectangular highlights the area where the EDX elemental map was collected; (**b**) phosphorus STEM-EDX map; (**c**) EDX spectrum corresponding to the map reported in **b**.

The spherical shape observed for the particles is probably an indication of damage, and it might be due to the probe sonication or energetic electron beam (200 kV) used for the acquisition of TEM images; the latter can cause degradation of the particles during the analysis [53] starting from their edges and determining the smoothed shape eventually observed [54].

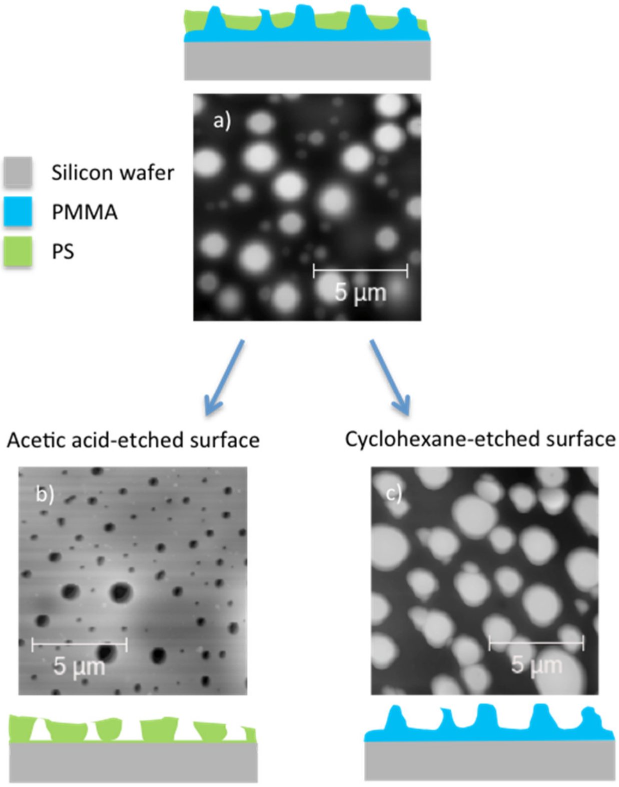

Figure S4. AFM topography of PS/PMMA blend, 5% wt./vol toluene, spin-cast film (**a**), acetic acid-etched film (**b**), and cyclohexane-etched film (**c**). Schemes illustrating the profile of surface topography are reported in the three cases.

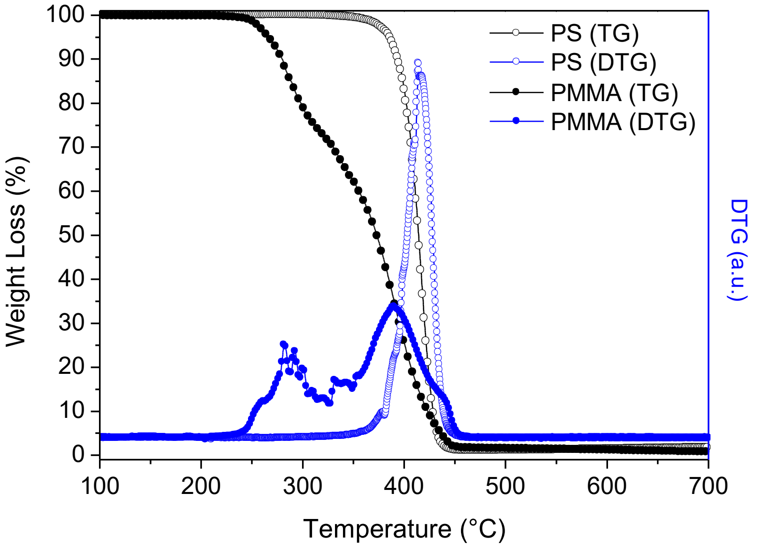

Figure S5. TG and DTG curves of PS and PMMA under nitrogen flow.

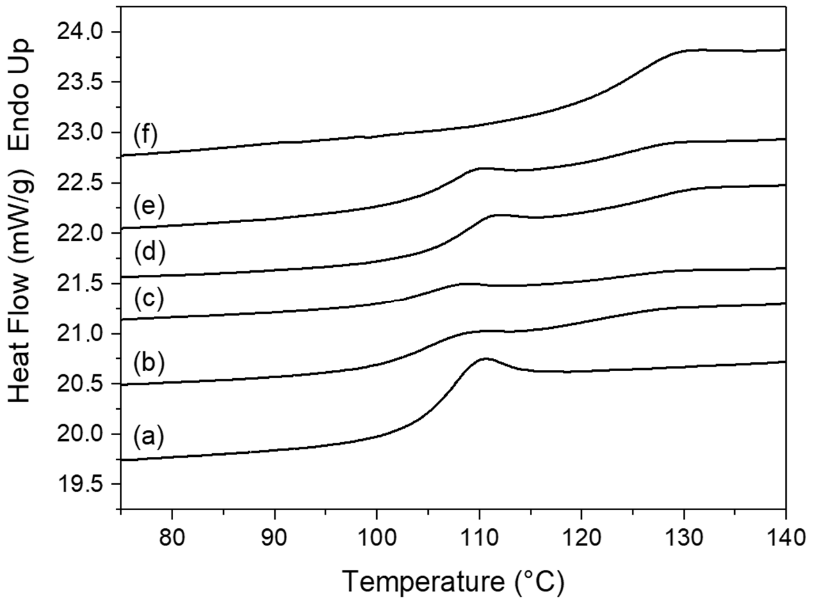

Figure S6. DSC curves second heating scan of PS (**a**), PS/PMMA (**b**), PS/PMMA/fl-bP0 (**c**), PS/PMMA/fl-bP3000 (**d**), PS/PMMA/fl-bP5000 (**e**), and PMMA (**f**).

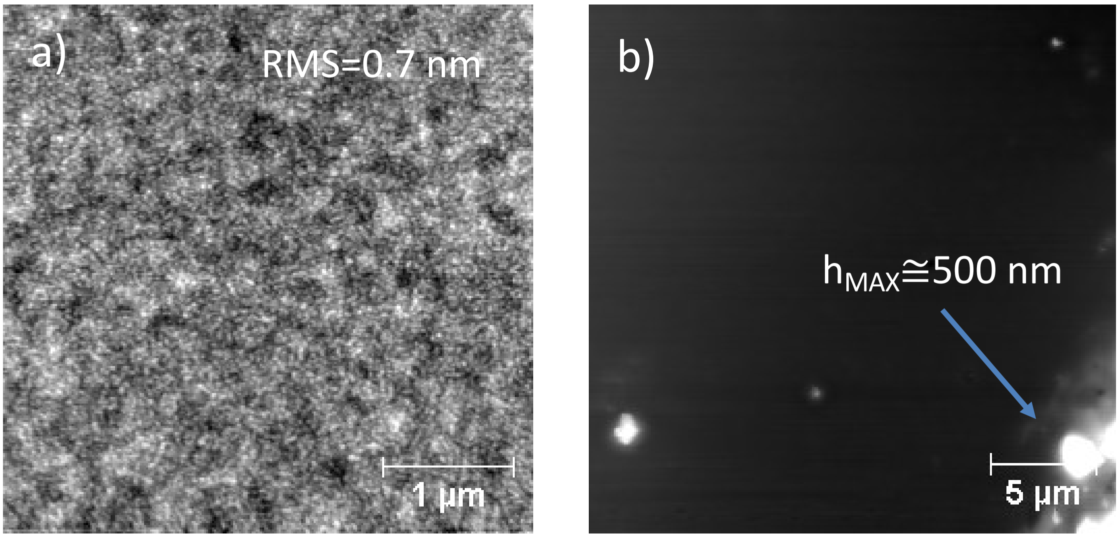

Figure S7. AFM topography of PMMA-*b*-PS (**a**) and PMMA-*b*-PS/fl-bP (**b**) 5% wt./vol toluene, as spin-cast.

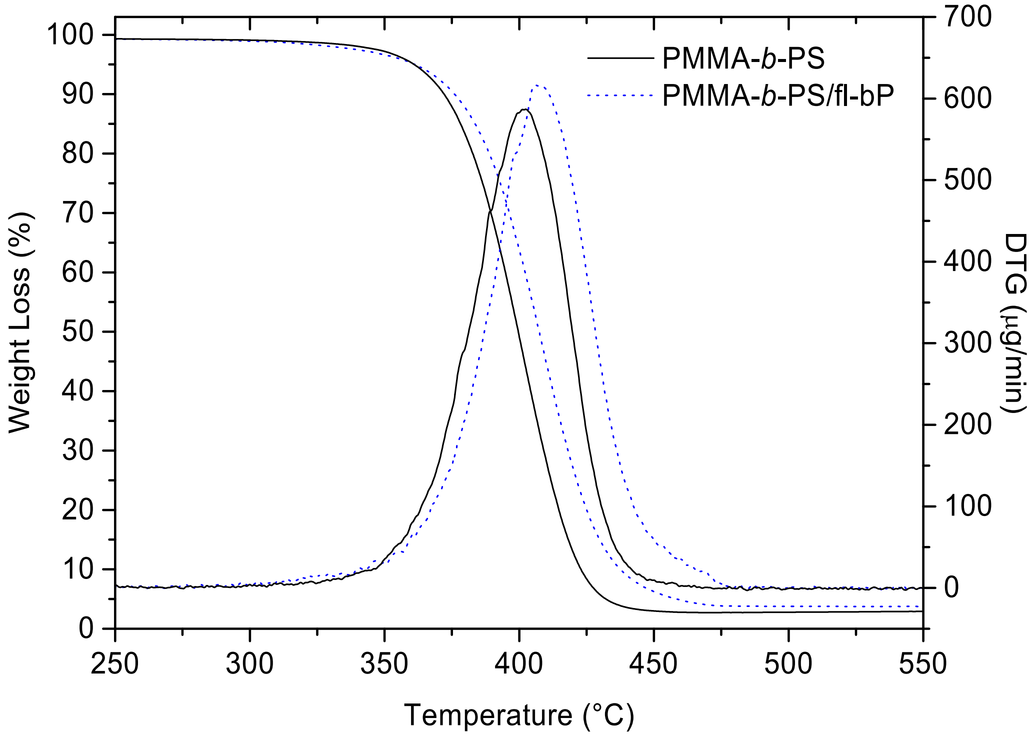

Figure S8. TG and DTG curves of PMMA-*b*-PS and PMMA-*b*-PS/fl-bP under nitrogen flow.
